# The effect of the local administration of biological substances on the rate of orthodontic tooth movement: a systematic review of human studies

**DOI:** 10.1186/s40510-021-00349-5

**Published:** 2021-02-01

**Authors:** Sarah Abu Arqub, Vaibhav Gandhi, Marissa G. Iverson, Maram Ahmed, Chia-Ling Kuo, Jinjian Mu, Eliane Dutra, Flavio Uribe

**Affiliations:** 1 Division of Orthodontics, University of Connecticut Health, 263 Farmington Ave, Farmington, CT 06032 USA; 2 L.M. Stowe Library, University of Connecticut Health, Farmington, CT USA; 3 Division of Orthodontics, University of Boston, Boston, MA USA; 4 Connecticut Convergence Institute for Translation in Regenerative Engineering, University of Connecticut Health, Farmington, CT USA

**Keywords:** Orthodontic tooth movement, Acceleration, Biological agents, Prostaglandins, Vitamin D, Platelet-rich plasma, Vitamin C, Relaxin, Human trials

## Abstract

**Background:**

The influence of different biological agents on the rate of orthodontic tooth movement (OTM) has been extensively reviewed in animal studies with conflicting results. These findings cannot be extrapolated from animals to humans. Therefore, we aimed to systematically investigate the most up-to-date available evidence of human studies regarding the effect of the administration of different biological substances on the rate of orthodontic tooth movement.

**Methods:**

A total of 8 databases were searched until the 16th of June 2020 without restrictions. Controlled randomized and non-randomized human clinical studies assessing the effect of biological substances on the rate of OTM were included. ROBINS-I and the Cochrane Risk of Bias tools were used. Reporting of this review was based on the Preferred Reporting Items for Systematic Reviews and Meta-Analyses (PRISMA) guidelines.

**Results:**

A total of 11 studies (6 randomized clinical trials and 5 prospective clinical trials) were identified for inclusion. Local injections of prostaglandin E1 and vitamin C exerted a positive influence on the rate of OTM; vitamin D showed variable effects. The use of platelet-rich plasma and its derivatives showed inconsistent results, while the local use of human relaxin hormone showed no significant effects on the rate of OTM.

**Limitations:**

The limited and variable observation periods after the administration of the biological substances, the high and medium risk of bias assessment for some included studies, the variable concentrations of the assessed biological agents, the different experimental designs and teeth evaluated, and the variety of measurement tools have hampered the quantitative assessment of the results as originally planned.

**Conclusions and implications:**

Despite the methodological limitations of the included studies, this systematic review provides an important overview of the effects of a variety of biological agents on the rate of tooth movement and elucidates the deficiencies in the clinical studies that have been conducted so far to evaluate the effectiveness of these agents in humans, providing some guidelines for future robust research.

**Trial registration:**

PROSPERO (CRD42020168481, www.crd.york.ac.uk/prospero)

## Background

Orthodontic tooth movement (OTM) occurs because of mechanical stimulus sequenced by remodeling of the alveolar bone and periodontal ligament (PDL). Bone remodeling is a process of bone resorption on the pressure sites and bone formation on the tension sites [1]. Forces applied to the teeth cause a local alteration in blood flow which in turn leads to the release of different inflammatory mediators such as cytokines, growth factors, neurotransmitters, colony-stimulating factors, and arachidonic acid metabolites [2, 3]. These in turn play an integral role in bone remodeling and subsequently cause tooth movement [3].

Orthodontic research has been heavily focused on accelerating the rate of tooth movement due to high demands from patients and clinicians [4]. Lengthy treatment duration requires a long commitment by patients and may lead to possible irreversible side effects such as external apical root resorption, white spot lesions, and dental caries [4–7]. Approaches that have been attempted to enhance the rate of (OTM) can be broadly categorized as biological, biomechanical, physical, and surgical [8–10].

Several animal studies have assessed the influence of biological substances on the rate of OTM, demonstrating favorable results [11]. Prostaglandins (PG) were among the first and most evaluated biological agents for accelerated OTM [12, 13]. Human relaxin hormone (HRH) has shown to decrease periodontal ligament organization in rats [14, 15], but yielded conflicting results in terms of its effects on OTM [16, 17]. Vitamin D (Vit D) was also studied in animals and demonstrated an increase in the number of osteoclasts, leading to acceleration of tooth movement [18]. In addition, vitamin C (Vit C) [19] and platelet-rich plasma (PRP) [20, 21] have also shown to increase the rate of OTM in animal models. However, animal data does not permit direct inferences to human scenarios [22, 23].

In consequence, it is not clear which substances may significantly affect clinical practice, and despite the promising results in animal studies, human prospective studies have reported conflicting views on the efficacy of biological substances on the acceleration of OTM [24–26]. The local administration of PG appeared to accelerate the rate of experimental tooth movement clinically [25, 27]. While the local administration of HRH showed no effect on the rate of tooth movement [28].The administration of other agents like Vit D showed an acceleratory response in a dose-dependent manner in one trial [26] and a decreased rate in orthodontic tooth movement in another [29]. In addition, PRP has shown effects that varied between studies [30–32].

### Objective

This systematic review aimed to provide an overview of the most used biological agents to accelerate tooth movement that has been experimented clinically in the literature and to describe their effectiveness. Additionally, the goal was to critically evaluate the experimental methodologies and outcome assessment methods of these studies.

## Materials and methods

### Protocol and registration

The guidelines in the Preferred Reporting Items for Systematic review and Meta-Analysis Protocols (PRISMA-P) statement [33] were used to develop a protocol that was registered in PROSPERO (CRD42020168481) [34]. The present systematic review was conducted according to the guidelines of the Cochrane Handbook for Systematic Reviews of Interventions version 6 [35].

### Eligibility criteria

The eligibility criteria for the participants, intervention, comparison, outcomes, and study design domains (PICOS) are presented in Table 1. We reviewed experimental prospective controlled studies (randomized and non-randomized) involving healthy patients undergoing active orthodontic treatment. The rate of tooth movement had to be investigated after the administration of the biological substance. Comparisons were made to the placebo intervention, no administration, or different dosages of the investigated substance. Studies comparing different biological substances without the presence of a placebo or no administration group, non-comparative studies, reviews, systematic reviews, and meta-analyses were excluded.

**Table 1:** Eligibility criteria for the present systematic review

Domain	Inclusion criteria	Exclusion criteria
**Participants**	Healthy human subjects undergoing any kind of active orthodontic treatment with orthodontic appliances.	• Animal subjects undergoing any kind of orthodontic tooth movement. • Human subjects after the cessation of active orthodontic tooth movement, or unhealthy human subjects suffering from syndromes or systematic diseases.
**Interventions**	Local or systemic administration of common biological agents (growth hormone, prostaglandins, parathyroid hormone, thyroxine, relaxin, vitamin D, platelet-rich plasma) to accelerate the rate of OTM	• Studies where the active substance or another intervention was used to decelerate OTM. • The use of other interventions to accelerate OTM including (surgical interventions, e.g. corticotomies, micro-osteoperforations, piezocisions, the use of low-level energy laser and vibration) ▪ The local or systemic administration of drugs that are manufactured through chemical synthesis by combining specific chemical ingredients which are not considered biologics and might include bisphosphonates, non-steroidal anti-inflammatory drugs (NSAIDS), immunosuppressants, anti-cancerous drugs and anticonvulsants.
**Comparisons**	Placebo intervention (preferably) or no intervention or administration of different dosages of the investigated substance.	
**Outcomes**	Qualitative and quantitative data if possible regarding the rate of orthodontic tooth movement (i.e. the amount of tooth movement in a specific period of time) measured by various ways (callipers, feeler gauges, lateral cephalometric or panoramic radiographs, cone beam computerized tomography, digital or stone study models, etc.).	
**Study design**	Experimental prospective controlled studies (randomized and non-randomized)	▪ Non-comparative studies. ▪ In vitro or ex vivo studies. ▪ Case reports, reviews, systematic reviews, and meta-analyses, case series, animal studies, opinion articles, and letters from editor

### Information sources and search strategy

A health sciences librarian searched the whole content of 8 databases (that included grey literature) until the 16th of June 2020. The strategies were developed by the health sciences librarian and were based on the MEDLINE search Table 2. No restrictions were imposed (status or date of publication and language). The reference lists of the included and excluded studies, the retrieved reviews, and other relevant articles were searched also.

**Table 2:** Strategies for database searches (up to June 16^th^, 2020)

Database	Search strategy	Hits
**PubMed/Medline**	((“Tooth Movement Techniques”[Mesh] OR “tooth movement”[tw] OR orthodont*[tw]) AND (“Cholecalciferol”[Mesh] OR “Growth Hormone”[Mesh] OR “growth hormone”[tw] OR “Parathyroid Hormone”[Mesh] OR “parathyroid hormone”[tw] OR PTH[tw] OR “Platelet-Rich Plasma”[Mesh] OR “platelet-rich plasma”[tw] OR PRP[tw] OR “platelet-rich fibrin”[tw] OR “Prostaglandins”[Mesh] OR prostaglandin[tw] OR prostaglandin*[tw] OR “Relaxin”[Mesh] OR relaxin[tw] OR “Thyroid Hormones”[Mesh] OR “Vitamins”[Mesh] OR “vitamin D”[tw] OR vitamin[tw] OR vitamin*[tw] OR calcitonin[tw] OR calcitriol[tw] OR tyrosine[tw])) NOT (“Animals”[Mesh] NOT (“Animals”[Mesh] AND “Humans”[Mesh]))	**307**
**Scopus (Elsevier)**	TITLE-ABS-Key((“tooth movement” OR orthodont*) AND (cholecalciferol OR “growth hormone” OR “parathyroid hormone” OR PTH OR “platelet-rich plasma” OR PRP OR “platelet-rich fibrin” OR prostaglandin OR prostaglandin* OR relaxin OR “thyroid hormone” OR “vitamin D” OR vitamin OR vitamin* OR calcitonin OR calcitriol OR *tyrosine))	**594**
**Cochrane Central Register of Controlled Trials (CENTRAL) (Wiley)**	All Text: (“tooth movement” OR orthodont*) AND (cholecalciferol OR “growth hormone” OR “parathyroid hormone” OR PTH OR “platelet-rich plasma” OR PRP OR “platelet-rich fibrin” OR prostaglandin OR prostaglandin* OR relaxin OR “thyroid hormone” OR “vitamin D” OR vitamin OR vitamin* OR calcitonin OR calcitriol OR *tyrosine)	**69**
**CINAHL (Ebsco)**	(MH Orthodontics+ OR TX “tooth movement” OR TX orthodont*) AND (MH Cholecalciferol OR TX cholecalcifereol OR MH Human Growth Hormone OR TX “growth hormone” OR MH Parathyroid Hormones+ OR TX “parathyroid hormone” OR TX PTH OR MH Platelet-Rich Plasma+ OR TX “platelet-rich plasma” OR TX PRP OR TX “platelet-rich fibrin” OR MH Prostaglandins+ OR TX prostaglandin OR TX prostaglandin* OR TX relaxin OR MH Thyroid Hormones+ OR TX “thyroid hormone” OR MH Vitamins+ OR TX “vitamin D” OR TX vitamin OR TX vitamin* OR MH Calcitonin OR TX calcitonin OR MH Calcitriol OR TX calcitriol OR MH Tyrosine OR TX *tyrosine) Limit to: Human	**140**
**Global Index Medicus (World Health Organization)**	(“tooth movement” OR orthodont*) AND (cholecalciferol OR “growth hormone” OR “parathyroid hormone” OR PTH OR “platelet-rich plasma” OR PRP OR “platelet-rich fibrin” OR prostaglandin OR prostaglandin* OR relaxin OR “thyroid hormone” OR “vitamin D” OR vitamin OR vitamin* OR calcitonin OR calcitriol OR *tyrosine)	**103**
**Dissertation Abstracts (ProQuest)**	ab((“tooth movement” OR orthodont*) AND (cholecalciferol OR “growth hormone” OR “parathyroid hormone” OR PTH OR “platelet-rich plasma” OR PRP OR “platelet-rich fibrin” OR prostaglandin OR prostaglandin* OR relaxin OR “thyroid hormone” OR “vitamin D” OR vitamin OR vitamin* OR calcitonin OR calcitriol OR tyrosine)) OR ti((“tooth movement” OR orthodont*) AND (cholecalciferol OR “growth hormone” OR “parathyroid hormone” OR PTH OR “platelet-rich plasma” OR PRP OR “platelet-rich fibrin” OR prostaglandin OR prostaglandin* OR relaxin OR “thyroid hormone” OR “vitamin D” OR vitamin OR vitamin* OR calcitonin OR calcitriol OR tyrosine)) OR su((“tooth movement” OR orthodont*) AND (cholecalciferol OR “growth hormone” OR “parathyroid hormone” OR PTH OR “platelet-rich plasma” OR PRP OR “platelet-rich fibrin” OR prostaglandin OR prostaglandin* OR relaxin OR “thyroid hormone” OR “vitamin D” OR vitamin OR vitamin* OR calcitonin OR calcitriol OR tyrosine)) OR diskw((“tooth movement” OR orthodont*) AND (cholecalciferol OR “growth hormone” OR “parathyroid hormone” OR PTH OR “platelet-rich plasma” OR PRP OR “platelet-rich fibrin” OR prostaglandin OR prostaglandin* OR relaxin OR “thyroid hormone” OR “vitamin D” OR vitamin OR vitamin* OR calcitonin OR calcitriol OR tyrosine))	**17**
**ClinicalTrials.gov**	Condition/disease: Orthodontic OR “Tooth Movement” Other Terms: cholecalciferol OR “growth hormone” OR “parathyroid hormone” OR PTH OR “platelet-rich plasma” OR PRP OR “platelet-rich fibrin” OR prostaglandin OR relaxin OR “thyroid hormone” OR vitamin OR calcitonin OR calcitriol OR tyrosine	**4**
**ISRCTN Registry**	(“tooth movement” OR orthodont*) AND (cholecalciferol OR “growth hormone” OR “parathyroid hormone” OR PTH OR “platelet-rich plasma” OR PRP OR “platelet-rich fibrin” OR prostaglandin OR prostaglandin* OR relaxin OR “thyroid hormone” OR “vitamin D” OR vitamin OR vitamin* OR calcitonin OR calcitriol OR *tyrosine)	**0**

### Study selection

The titles and abstracts of the retrieved records were assessed independently and in duplicate, for inclusion, by two authors (AS and AM). The same procedure was repeated for the full text of potentially included studies. Author (GV) settled any disagreements, and records of the decisions were kept. The extent of agreement between assessors was not calculated with kappa statistics as it is not recommended [35].

### Data collection and data items

Data extraction followed the previously described procedure. A customized data collection form was created and used to gather information from the selected studies. This information included authors, year of publication, type of studies, details of the interventions, characteristics of participants, duration of treatment, and outcome measures.

### Risk of bias in individual studies

The ROBINS-I for non-randomized [36] and the Cochrane Risk of Bias Tool for randomized studies [37] were used to assess the risk of bias using the same procedures. Summaries of the risk of bias within the study were produced by adhering to the Higgins et al. [35] approach.

### Summary measures and synthesis of results

As an adequate amount of information and number of studies regarding each of the studied biological agents were not retrieved, quantitative synthesis of results was not carried out, although this was originally planned [35, 38].

### Risk of bias across studies and additional analyses

Although pre-planned, analyses for “small-study effects” and publication bias, as well as exploratory subgroup analyses, were not conducted because we could not identify a sufficient number of studies [37] .The GRADE approach (Grading of Recommendation, Assessment, Development and Evaluation) was used to appraise the quality of evidence [39].

## Results

### Study selection

**Figure 1:**
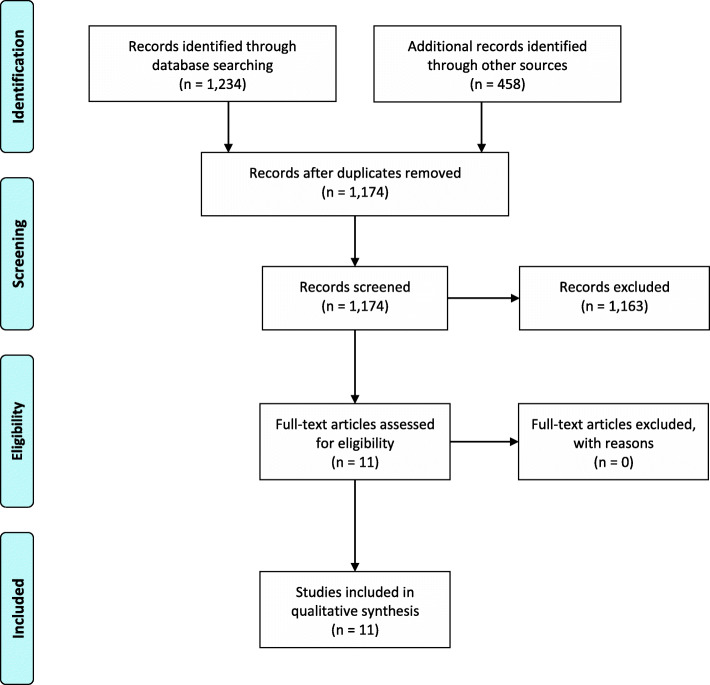
PRISMA

Initially, 1234 records were identified through database searching, and 458 additional records were identified through different databases. One thousand one hundred seventy-four records resulted after the removal of the duplicates; they were screened, and a total of 11 studies were identified for inclusion in the systematic review (Fig. 1).

### Study characteristics

Table 3 presents the characteristics of the studies included. Out of 11 studies, three tested PG [25, 27, 40], three tested Vit D [26, 41, 43], one examined HRH [28], one examined leukocyte platelet-rich fibrin (LPRF) [31], one examined platelet-rich fibrin (PRF) [30], one examined PRP [32], and one examined Vit C [42]. Six were randomized controlled trials (RCT) [26, 28, 31, 32, 42, 43] including four studies with split mouth design [26, 31, 32, 43]. Five studies [25, 27, 30, 40, 41] were prospective clinical trials with split mouth study design. The described studies had experiments that lasted between 3 weeks (minimum observation period) [26] and 6 months (maximum observation period) [30]. All of them had male and female subjects in their sample population except for 2 studies that did not specify the gender [26, 43].

**Table 3:** Study characteristics: participants (sample size, demographic information), intervention (orthodontic treatment), observation, comparison (biological substance), dose and route of administration, outcome (rate of tooth movement), and study design

Study	Participants	Intervention	Observation/tool used for assessment	Comparison	Dose and route of administration: E	Outcome (tooth movement)
**Yamasaki et al.** **[**27**]** Prospective clinical trial (split mouth)	**Phase 1:** 9 patients; 8F, 1 M; (11.8 Y) **Phase 2:** 8 patients; 6F, 2 M (15.6 Y) E: 10; C: 10 (quadrants) **Phase 3:** 8; 6F, 2 M (12.9 Y) E: 10; C:10 (quadrants)	FPm extraction cases **Phase 1:**Lingual arch + 2 double springs; 100 gm, constant force Follow-up period: between 8 and 26 days **Phase 2:** closing loop; 150 gm, constant force Follow-up period: Between 10 days and 3 weeks. **Phase 3:** Open coil springs; 150 gm, constant force Anchorage (HG or HA) Follow-up period: Between 1.5 and 4.8 months	**Phase 1**: Buccal movement of FPm **Phase 2**: Canine retraction **Phase 3:** Canine retraction Clinical measurement	**PGE1**	**Phase 1:** E: 10 μg; submucosal buccal right FPm Frequency: inconsistent (3 to 5 injections) **Phase 2:** E: 10 μg; submucosal distal to canine Frequency: inconsistent (3 to 4 injections) **Phase 3:** E: 10 μg; submucosal distal to canine Frequency: inconsistent (5 to 13 injections)	**mm/month** **Phase 1:** E/C ratio: 2.14 ± 0.33 **Phase 3:** E: 2.07 ± 0.26 C: 1.30 ± 0.16
**Spielmann et al.** **[**25**]** Prospective clinical trial (split mouth)	5 Patients; 1 F, 4 M (16 Y)	Elastic chain between FPm (transversely) Force application: 4 weeks (weekly reactivation) Follow-up period: month	Lingual movement of FPm Photographs	**PGE1**	E: (10 μg); local administration; weekly, (for 4 weeks)	**mm/month** E: 3.10 C: 1.03
**Patil et al.** **[**40**]** Prospective clinical trial (split mouth)	14 Patients; 10 F, 4 M (17.7 Y)	FPm extraction cases, NiTi retraction springs; 150 g Anchorage: TPA and 2nd molars Follow-up period: 2 months	Canine retraction Occlusogram	**PGE1**	E: (1 g); distal to canine; (days 1, 6, and 17)	**mm/2 months** E: 3.48 ± 0.69 C: 2.01 ± 0.49
**Al-Hasani et al.** **[**26**]** RCT (split mouth)	15 Patients; (17–28 Y) E1: 5; E2: 5; E3: 5 (quadrants) C: 15 (quadrants)	FPm extraction orthodontic cases 150 g retraction force Anchorage: TPA, stops, ligating SPm and FM Follow-up period: 3 weeks	Canine retraction Clinical measurements	**Vitamin D (calcitriol)**	E1: 15 pg, E2: 25 pg, E3: 40 pg vit D; local administration; weekly (for 3 weeks)	**mm/3 weeks** E1: 1.29 ± 0.61 C1: 1.42 ± 0.63 E2: 1.57 ± 0.84 C2: 1.04 ± 0.33 E3: 1.15 ± 0.36 C3: 1.04 ± 0.3
**McGorray et al.** **[**28**]** RCT	39 Patients; E: 16F, 4 M (26.2 Y) C: 12F, 7 M (27.7 Y)	Aligner therapy: 4 maxillary aligners (2 weeks/aligners) (0.5 mm anteroposterior movement) Follow-up period: 2 months	Right or left central incisor (crown tipping) Superimposition digital	**Human relaxin hormone**	E: (50 μg/0.2 ml); 2 injections (facial and lingual); local infiltration; weekly, (for 8 weeks)	**mm/2 months** E: 0.83 C: 0.83
**Ciur et al.** **[**41**]** Prospective clinical trial (split mouth)	6 Patients; 3F, 3 M (18 Y)	FPm extraction cases Closed coil NiTi spring; 150 g Anchorage:TPA Follow-up period: 3 weeks	Canine retraction CBCT	**Vitamin D3 (decostriol)**	E: (42 pg/1 ml) 0.2 mL vit D; local administration; weekly (for 3 weeks)	**mm/month** E: 1.7 ± 0.63 C: 1.00 ± 0.61
**Tehranchi et al.** **[**31**]** RCT (split mouth)	8 Patients; 3F, 5 M (17.37 Y). 30 extraction sockets (E: 15, C: 15)	FPm extraction cases Closed coil NiTi spring; 150 g Follow-up period: 4 months	Canine retraction Stone dental casts-digital caliper	**LPRF**	E: LPRF immediate placement in extraction socket; (only once)	**mm/4 months** E: 6.65 ± 0.83 C: 6.76 ± 0.76
**Nemtoi et el** **[**30**].** Prospective clinical trial (split mouth)	20 patients; 11F, 9 M (16.43 Y) 40 extraction sockets (E: 20, C: 20)	FPm extraction cases Closed coil NiTi spring; 150 g Follow-up period: 6 months	Canine retraction Stone dental casts-ruler	**PRF**	E: PRF plugs in extraction socket; (only once)	**mm/6 months** E:3.1 C:1.9
**Yussif et al.** **[**42**]** RCT	12 Patients; 9 F, 3 M (16–34 Y) E: 6 subjects; C:6 subjects	Unilateral palatal impacted max canine Elastic chain traction (every 2 weeks) Follow-up period: 1 month	Canine traction Clinically on radiographs (panoramic and occlusal)	**Vitamin C**	E: (200–300 mg); Intraepidermal injection; 1–1.5 mL of l-ascorbic acid divided by 6 to determine dose for 1 tooth: biweekly (for 6–8 continues orthodontic visits)	**mm/month** E: 2.25 ± 0.274 C: 1.08 ± 0.376
**Varughese et al.** **[**43**]** RCT, (split mouth)	15 Patients; (22.5 Y)	FPm extraction cases Closed coil NiTi spring; 150 g Anchorage: bilateral 2nd molar banding/TPA Follow-up period: 3 months	Canine retraction Stone dental casts-digital caliper	**Vitamin D (1,25 DHC)**	E: 50 pg per 0.2 mL; intraligamentary injection; distal to the canine; monthly (for 12 weeks)	**1st month** E: 1.568 ± 0.368 C: 1.0260 ± 0.1777
**El-Timamy et al.** **[**32**]** RCT, (split mouth)	15 Patients; 15 F (18 ± 3 Y)	FPm extraction cases Closed coil NiTi spring; 150 g Anchorage: mini-implants (TADs) Follow-up period: 4 months	Canine retraction Digital dental casts—superimposition	**PRP**	E: 250 mg (0.25 mL) PRP + 10% CaCl2 solution; intraligamentary; every 3 weeks (at 0, 3, 6 weeks)	**mm/4 months** E:4.57 ± 1.13 C:4.53 ± 1.12

*E* experimental, *C* controls, *M* males, *F* females, *L* load, *Y* years, *W* weeks, *TPA* trans-palatal arch, *HG* headgear, *HA* holding arch, *PGE1* prostaglandin, *LPRF* leukocyte platelet-rich fibrin, *PRF* platelet-rich fibrin, *PRP* platelet-rich plasma, *1,25 DHC* 1,25-dihydroxycholecalciferol, *FPm* first premolar, *SPm* second premolar, *FM* force module, *CBCT* cone beam computed tomography

Mean age of the included subjects ranged between 11.8 [27] and 34 years old [42]. Sample size calculation was conducted in only 3 studies [30–32]. Malocclusion was not specified in the majority of the studies [25, 27, 30–32, 40, 41]. Coil springs and elastomeric chains were used for canine retraction [26, 30–32, 40, 41, 43], elastomeric chains extended between the lingual surfaces of the opposing upper first premolars [25] and lingual arches with soldered double springs [27] were used for transverse premolar movements, and clear aligners were used in one study for incisor alignment [28]. The rate of tooth movement was assessed on stone casts [30, 31, 43], digital casts superimpositions [28, 32], cone beam computed tomography scans [41], occlusograms [40], photographs [25], direct clinical assessment [26, 27], and clinical evaluation using panoramic and occlusal radiographs [42].

### Risk of bias within studies

Tables 4 and 5 contain a summary of the risk of bias assessment. Three of the RCTs [26, 31, 42] were assessed as having some concerns in their overall risk of bias, mainly due to the lack of the concealed allocation in the randomization process. As for the non-randomized clinical trials, the overall assessment for the risk of bias was serious for 2 studies [25, 27] and medium for the other three [30, 40, 41], indicating poor reporting and experimental design of these studies.

**Table 4:** Summary of risk of bias assessment for non-randomized studies-ROBINS-1 tool

Domain	Yamasaki et al. [27]	Spielmann et al. [25]	Patil et al. [40]	Ciur et al. [41]	Nemtoi et el [30].
**Bias due to confounding**	Serious	Serious	Moderate	Moderate	Moderate
**Bias in selection of participants for the study**	Serious	Moderate	Moderate	Moderate	Moderate
**Bias in classification of interventions**	Moderate	Low	Low	Low	Low
**Bias in measurement of outcomes**	Serious	Serious	Moderate	Moderate	Moderate
**Bias in selection of the reported result**	Moderate	Low	Low	Low	Low
**Overall**	Serious	Serious	Moderate	Moderate	Moderate

**Table 5:** Summary of risk of bias assessment for randomized studies—The Cochrane’s Collaboration’s tool

Domain	Al-Hasani et al. [26]	McGorray et al. [28]	Tehranchi et al. [31]	Yussif et al. [42]	Varughese et al. [43]	El-Timamy et al. [32]
**Randomization process**	High	Some concerns	Some concerns	Some concerns	Low	Low
**Deviations from intended interventions**	Some concerns	Low	Low	Some concerns	Low	Low
**Missing outcome data**	Low	Low	Some concerns	Low	Low	Low
**Measurement of the outcome**	Some concerns	Low	Low	Unclear	Some concerns	Some concerns
**Selection of the reported result**	Low	Low	Low	Low	Some concerns	Low
**overall**	Some concerns	Low	Some concerns	Some concerns	Low	Low

### Results of individual studies

**Figure 2:**
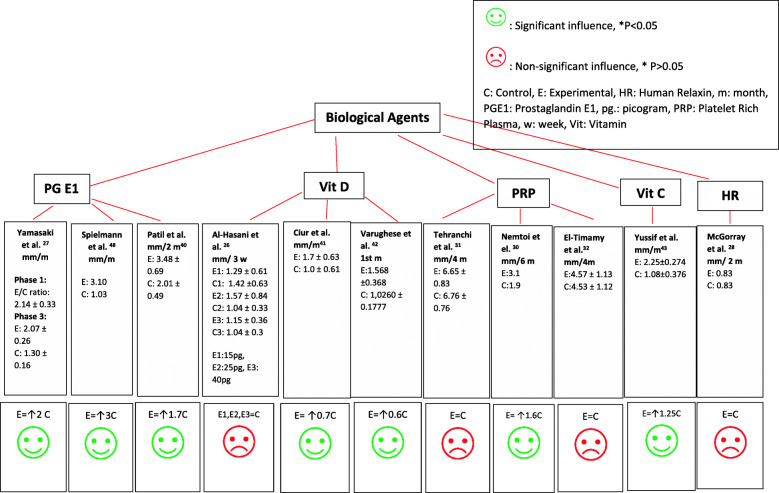
Effects of biologics

Figure 2 illustrates the overall clinical effects of administering the biological agents in all included experiments. Despite the different observation periods, concentrations used and frequency of injections, local administration of PGE1 was found to exert an increasing effect on the rate of OTM which was statistically significant (*P* < 0.05) in all included three studies [25, 27, 40]. Vit D showed conflicting results. Two studies reported a positive influence on the rate of OTM [41, 43]. On the other hand, one study which compared 3 different experimental groups, each using different concentrations of Vit D (15 pg, 25 pg, and 40 pg), showed non-significant effects in all three groups compared to the controls [26]. HRH showed no significant effect on accelerating tooth movement using aligners [28]. On the other hand, PRP derivatives showed positive effects in a study that utilized platelet-rich fibrin (PRF) in extraction sockets [30] and non-significant results in the other two [31, 32]. Vit C illustrated a positive influence on the rate of tooth movement when injected for canine retraction [42].

### Risk of bias across studies and additional analyses

Analyses for “small-study effects,” publication bias, or subgroup analyses were not possible as discussed previously [33]. Regarding the effect of the investigated biological agents on the rate of orthodontic tooth movement, the quality of available evidence as graded by the Grading of Recommendations Assessment**,** Development and Evaluation (GRADE) approach using the GRADEpro Guideline Development Tool (Software) [44] (i.e., the confidence that we have that the true effect is more or less similar to the estimated effect) ranged between moderate (Vit D and C studies) to high (HRH and PRP studies) for the RCTs, mainly because of the lack of allocation concealment which introduced a selection bias to the trials graded as moderate. Concerns regarding the small sample size, inconsistency of the interventions and outcomes, and high risk of bias led further to downgrading the quality of evidence for the non-randomized controlled trials. This quality of evidence ranged between low and moderate in the final assessment of the confidence in the observed estimates (Tables 6 and 7).

**Table 6:** Quality of the available evidence on the rate of orthodontic tooth movement for the non-randomized trials

Quality assessment						Effect	Quality
Studies	Risk of bias	Inconsistency	Indirectness	Imprecision	Other		
**PGE1**							
3	Serious^a^	Not serious	Not serious	Not serious	None	Increase in the EG	⨁⨁⨁◯^b^ **Moderate**
**Vit D**							
1	Serious^a^	Serious	Not serious	Not serious	None	Increased in EG	⨁⨁◯◯^b^ **Low**
**PRF**							
1	Not serious	Not serious	Not serious	Serious	None	Increased in EG	⨁⨁⨁◯^b^ **Moderate**

*EG* experimental group, *PGE1* prostaglandin E1, *PRF* platelet-rich fibrin, *Vit D3* vitamin D

^a^The results are based on few studies with small sample size

^b^Owing to the non-randomized study design (prospective non-randomized trials), high risk of bias, the respective grading of evidence ranged between moderate and low. Possible serious risk because of the multitude of domains rated as unclear and the inconsistency in the measurement techniques to evaluate the rate of OTM

**Table 7:** Quality of available evidence on the rate of orthodontic tooth movement for the RCT

Quality assessment						Effect	Quality
Studies	Risk of bias	Inconsistency	Indirectness	Imprecision	Other		
**Vit D**							
2	Serious^a^	Not serious	Not serious	Not serious^a^	None	Al-Hasani [26]: no effect Varughese [43]: increased in the EG	⨁⨁⨁◯^b^ **Moderate**
**HRH**							
1	Not Serious	Not serious	Not serious	Not serious	None	No difference	⨁⨁⨁⨁ **High**
**PRP**							
1	Not serious	Not serious	Not serious	Not serious	None	Tehranchi [31]: no effect El-Timamy [32]: no effect	⨁⨁⨁⨁ **High**
**Vit C**							
1	Serious^a^	Not serious	Not serious	not serious	None	Increased in the EG	⨁⨁⨁◯^b^ **Moderate**

*EG* experimental group, *HRH* human relaxin hormone, *PRP* platelet-rich plasma, *Vit C* vitamin C, *Vit D* vitamin D

^a^The results overall are based only on few studies

^b^Owing to the lack of concealed allocation in the randomization, the respective grading of evidence started as moderate

## Discussion

### Summary of evidence

Orthodontic tooth movement has been defined as “the result of a biologic response to interference in the physiologic equilibrium of the dentofacial complex by an externally applied force [45].” The sequence of cellular, molecular, and tissue-reaction events during orthodontic tooth movement has been extensively studied [46]. Several factors, alone or in combination, might influence remodeling activities and ultimately tooth displacement [1], and the alterations in bone turnover and density may affect the rate of movement. In this sense, many biological agents play a role in the inflammatory process and alter the pathways related to bone remodeling that accompanies OTM [45]. However, direct inference of information derived from animal experiments to human clinical settings may not be made [17–21]. Hence, this systematic review evaluated whether locally administered biological substances such as PG, HRH, Vit D, Vit C, PRP, and its derivatives can significantly accelerate OTM in humans.

On the basis of the collected studies, a variable effect on the rate of movement was detected between the different biological agents, with PG [25, 27, 40] and Vit C [42] causing a positive influence, HRH showing no influence [28], and Vit D and PRP and its derivatives exerting a variable dose-dependent influence [26] (Fig. 2). The set of retrieved data is limited, and the level of confidence in the observed estimates was deemed to be variable due to the limited number of studies that have assessed each agent, small sample sizes, different age groups, different appliances for tooth movement and methods of magnitude of tooth movement assessment, the high risk of bias for some of the investigations, and the different observational periods and frequencies of application for the biological agents.

Among the studied biological agents, PGs are the most significant in OTM acceleration as they stimulate both osteoclasts and osteoblasts. These PGs also elevate metalloproteinases’ levels and lead to decrease in the production of procollagen which is essential for bone and periodontal ligament remodeling [47]. Although the local administration of PG in the three non-randomized clinical trials included in this systematic review was found to exert an increasing effect on the rate of OTM by 1–2 mm per month [25, 27, 40], the results of the GRADE assessment revealed an overall certainty of evidence being moderate suggesting that the true effect could be markedly different from the estimated effect. This might be because these studies are non-randomized with small sample sizes and short observation periods that ranged between 1 [25, 27] and 2 months [40].

On the other hand, local injections of calcitriol, a Vit D metabolite, were shown to exert variable effects on the rate of OTM, but with a low to moderate level of confidence. Vit D plays an important role in calcium homeostasis with calcitonin and parathyroid hormone (PTH) and constitutes a potent modulator of bone metabolism [24]. The conflicting results between the studies on the effect of Vit D on accelerating tooth movement might be attributed to the fact that these studies utilized different concentrations for Vit D, namely 42 pg [41] and 50 pg [43]. Furthermore, Al-Hasani et al. [26] compared 3 different concentrations in the same study (15 pg, 25 pg, and 40 pg). Thus, there may be a differential impact of Vit D depending on the dose [47]. More importantly, the effects of Vit D on bone turnover depend on the stage of osteoblast differentiation [48]. It has been reported that normal levels of Vit D act via the vitamin D receptor (VDR) in mature osteoblasts, decreasing the receptor activator of nuclear factor kappa-Β ligand (RANKL)/osteoprotegerin (OPG) ratio and leading to reduction of osteoclastic bone resorption. Similarly, Vit D acts in mature osteoblasts increasing bone formation rate [24]. However, increased levels of Vit D act in less-mature osteoblasts elevating the RANKL/OPG ratio, thus stimulating osteoclastic bone resorption [49, 50]. Studies of conditional deletion of the VDR from the osteoblast lineage suggest that early osteoblastic cells may mediate an increase in bone resorption induced by Vit D [51]. Thus, the effect of Vit D is related to increasing the expression of RANKL by local cells and therefore activation of osteoclasts [52].

HRH is a peptide with strong effects on collagen turnover. It has been shown that it increases collagen in the tension sites and decreases it in the compression sites during orthodontic tooth movement [53, 54]. Its effects on remodeling soft tissue and on regulation of several mediators that stimulate osteoclast formation increased the attention of researchers in orthodontics [53]. Based on the findings of the present review, only one RCT assessed the local administration of HRH in humans [28]. It was not found to have any statistically significant effect on the rate of OTM. The GRADE assessment suggested that this estimate is close to the true effect. But the authors of this study suggested that the used local doses of HRH might have been too low to affect tooth movement. Previous studies on animals have produced contradictory outcomes. Madan et al. [14] measured the effect of HRH on orthodontic tooth movement and periodontal ligament (PDL) structures in rats and concluded that it does not accelerate OTM, although it can reduce the level of PDL organization and mechanical strength and increase tooth mobility at early time points. On the other hand, Liu et al. [17] administered HRH to young rats through either minipumps or subcutaneous injections and concluded that it may accelerate the early stages of orthodontic tooth movement in rats.

Concerning the local administration of PRP and its derivatives, only one non-randomized clinical trial that used PRF in the extraction sockets [30] showed significant increase in the rate of OTM. The other two RCTs, one that used PRP concentrate [32] and another that investigated LPRF [31], showed no significant effect on the rate of OTM. The different effects might be attributed to the different concentrations and delivery methods such as injections [32] and plugs in the extraction sockets [30, 31]; the various PRP presentations (PRF [30], LPRF [31], and PRP [32]); and the different observation periods (6 months for the PRF [30], 4 months for the LRPF [31], and 6 weeks for the PRP [32]). In a recent review, it was shown that the differences in the methods to create and activate PRP, the variable platelet concentrations used in different clinical studies, and different delivery methods make it impossible to directly compare clinical studies assessing the effects of PRP [55]. Regardless of the methodology, another issue with the use of PRP is the challenge of retaining PRP in a physiologically active state for long time, which mainly depends on the form of administration and leukocyte concentration [55, 56]. On the other hand, it has been illustrated that the combination of fibrins and cytokines within the PRF is a powerful bio-scaffold with an integrated reservoir of growth factors for tissue regeneration [57], which might be a reason why the rate of OTM was accelerated with the use of PRF [30] compared to the other 2 studies of PRP and its derivatives [31, 32]. The GRADE assessment suggested that this estimate is close to the true effect with high certainty of evidence for the RCTs and moderate certainty of evidence for the controlled trial.

The critical role of ascorbic acid (Vit C) in osteoclast stimulation in cell culture media has been confirmed in several investigations [58, 59]. The lack of Vit C halts osteogenesis and periodontal ligament organization [60]. Ascorbic acid deficiency inhibits degradation and regeneration of collagen fibers, which are important in orthodontic tooth movement [60]. Within the scope of this systematic review, a significant effect on the rate of canine traction resulted from the local injection of Vit C. However, the GRADE assessment suggested that this estimate is probably not close to the true effect, the risk of bias for this study was high, the sample size was small, and there were some concerns in the research methodology. Even though normal dietary ingestion of Vit C is needed for a healthy periodontium during orthodontics [61], it is still doubtful whether Vit C supplementation affects OTM clinically.

### Strengths and limitations

This systematic review followed the standard guidelines with a comprehensive search strategy that included pertinent records. The strategies for the database search were comprehensive and covered until June 2020. There was no language restriction, and all steps were carried out independently and in duplicate. Settling any disagreement was carried out after consulting a third author. Care was taken to minimize bias as much as possible.

On the other hand, some limitations became clear because of the experimental designs of the studies and the characteristics of the data used for the review. This led to a moderate average rating for the quality of evidence. The scarcity in the relevant information, the moderate to low risk of bias assessment to many studies, the use of different biological agents, and the different observation periods made it difficult to perform a meta-analysis and additional analyses, even though initially planned.

The application of the biological agents has certain limitations. Majority of these agents have short half-life; therefore, multiple applications of the agent are required, which is not practical in clinical orthodontics. Further, the potential side effects that might result from the application of these agents over an extended period must be taken into consideration. The limited observation period for the studies hindered the evaluation of the true effect and potential harm of these agents if applied over the entire length of the orthodontic treatment. Hence, it would be interesting to conduct clinical studies with an observation period encompassing the full length of treatment. This would provide a more meaningful conclusion and give insight to the specific reduction in treatment duration to justify any of these adjunct therapies.

Finally, the sample size calculation was not performed in majority of the studies which poses limitations in terms of accuracy of the results and increases the chance of a type 2 error.

## Conclusions

Based on the collected data, the local administration of the biological agents during orthodontic treatment may have different effects on the rate of tooth movement in humans. Although the assessed level of evidence reflects that these results should be regarded cautiously, the possible implications should not be ignored, and thorough clinical research is required to investigate their effect and efficacy for the entire length of orthodontic treatment.

## Data Availability

Not applicable.

## Abbreviations

LPRF: Leukocyte platelet-rich fibrin
OPG: Osteoprotegerin
OTM: Orthodontic tooth movement
PDL: Periodontal ligament
PG: Prostaglandins
PRP: Platelet-rich plasma
PRF: Platelet-rich fibrin
HRH: Human relaxin hormone
RANKL: Receptor activator of nuclear factor kappa-Β ligand
RCT: Randomized clinical trials
VDR: Vitamin D receptor
Vit D: Vitamin D
Vit C: Vitamin C
GRADE: Grading of Recommendations Assessment, Development and Evaluation

## References

[1] Davidovitch Z (1991). *Tooth movement*. Crit Rev Oral Biol Med.

[2] Meikle MC (2006). The tissue, cellular, and molecular regulation of orthodontic tooth movement: 100 years after Carl Sandstedt. Eur J Orthod.

[3] Davidovitch Z (1988). Neurotransmitters, cytokines, and the control of alveolar bone remodeling in orthodontics. Dent Clin N Am.

[4] Skidmore KJ (2006). Factors influencing treatment time in orthodontic patients. Am J Orthod Dentofacial Orthop.

[5] Jiang R-p, McDonald J, Fu M-k (2010). Root resorption before and after orthodontic treatment: a clinical study of contributory factors. Eur J Orthod.

[6] Pinto AS (2017). Gingival enlargement in orthodontic patients: effect of treatment duration. Am J Orthod Dentofacial Orthop.

[7] Richter AE (2011). Incidence of caries lesions among patients treated with comprehensive orthodontics. Am J Orthod Dentofacial Orthop.

[8] Long H (2013). Interventions for accelerating orthodontic tooth movement: a systematic review. Angle Orthod.

[9] Makrygiannakis MA, Kaklamanos EG, Athanasiou AE (2018). Does common prescription medication affect the rate of orthodontic tooth movement? A systematic review. Eur J Orthod.

[10] JP R (2013). Use of laser in orthodontics: applications and perspectives. Laser Ther.

[11] Santana LG (2020). Systematic review of biological therapy to accelerate orthodontic tooth movement in animals: Translational approach. Arch Oral Biol.

[12] Yamasaki K, Miura F, Suda T (1980). Prostaglandin as a mediator of bone resorption induced by experimental tooth movement in rats. J Dent Res.

[13] Yamasaki K, Shibata Y, Fukuhara T (1982). The effect of prostaglandins on experimental tooth movement in monkeys (Macaca fuscata). J Dent Res.

[14] Madan MS (2007). Effects of human relaxin on orthodontic tooth movement and periodontal ligaments in rats. Am J Orthod Dentofacial Orthop.

[15] Nicozisis JL, Nah-Cederquist HD, Tuncay OC (2000). Relaxin affects the dentofacial sutural tissues. Clin Orthod Res.

[16] Stewart DR (2005). Use of relaxin in orthodontics. Ann N Y Acad Sci.

[17] Liu ZJ (2005). Does human relaxin accelerate orthodontic tooth movement in rats?. Ann N Y Acad Sci.

[18] Collins MK, Sinclair PM (1988). The local use of vitamin D to increase the rate of orthodontic tooth movement. Am J Orthod Dentofacial Orthop.

[19] Miresmaeili A (2015). Effect of dietary vitamin C on orthodontic tooth movement in rats. J Dent (Tehran, Iran).

[20] Rashid A (2017). Effect of platelet-rich plasma on orthodontic tooth movement in dogs. Orthod Craniofac Res.

[21] Nakornnoi T, Leethanakul C, Samruajbenjakun B (2019). The influence of leukocyte-platelet-rich plasma on accelerated orthodontic tooth movement in rabbits. Korean J Orthod.

[22] Shanks N, Greek R, Greek J. Philosophy, ethics, and humanities in medicine. Philos Ethics Humanit Med. 2009;4(2). 10.1186/1747-5341-4-2 PMC2642860 19146696

[23] Güleç A (2017). Effects of local platelet-rich plasma injection on the rate of orthodontic tooth movement in a rat model: a histomorphometric study. Am J Orthod Dentofacial Orthop.

[24] Kale S (2004). Comparison of the effects of 1, 25 dihydroxycholecalciferol and prostaglandin E2 on orthodontic tooth movement. Am J Orthod Dentofacial Orthop.

[25] Spielmann T, Wieslander L, Hefti A (1989). Acceleration of orthodontically induced tooth movement through the local application of prostaglandin (PGE1). Schweiz Monatsschr Zahnmed.

[26] Al-Hasani NR (2011). Clinical efficacy of locally injected calcitriol in orthodontic tooth movement. Int J Pharm Pharm Sci.

[27] Yamasaki K (1984). Clinical application of prostaglandin E1 (PGE1) upon orthodontic tooth movement. Am J Orthod.

[28] McGorray SP (2012). A randomized, placebo-controlled clinical trial on the effects of recombinant human relaxin on tooth movement and short-term stability. Am J Orthod Dentofacial Orthop.

[29] Shetty A, et al. Local infiltration of vitamin D3 does not accelerate orthodontic tooth movement in humans: a preliminary study. Angle Orthod. 2015.

[30] Nemtoi A (2018). The effect of a plasma with platelet-rich fibrin in bone regeneration and on rate of orthodontic tooth movement in adolescents. Rev Chim.

[31] Tehranchi A (2018). The effect of autologous leukocyte platelet rich fibrin on the rate of orthodontic tooth movement: a prospective randomized clinical trial. Eur J Dent.

[32] El-Timamy A (2020). Effect of platelet-rich plasma on the rate of orthodontic tooth movement: a split-mouth randomized trial. Angle Orthod.

[33] Moher D (2015). Preferred reporting items for systematic review and meta-analysis protocols (PRISMA-P) 2015 statement. Syst Rev.

[34] Chien PF, Khan KS, Siassakos D (2012). Registration of systematic reviews: PROSPERO. BJOG.

[35] Higgins JP, et al. Cochrane handbook for systematic reviews of interventions: Wiley; 2019.

[36] Sterne JA, et al. ROBINS-I: a tool for assessing risk of bias in non-randomised studies of interventions. Bmj. 2016;355. 10.1136/bmj.i4919 PMC5062054 27733354

[37] Higgins JP (2011). The Cochrane Collaboration’s tool for assessing risk of bias in randomised trials. Bmj.

[38] Borenstein M, et al. Introduction to meta-analysis: Wiley; 2011.

[39] Guyatt GH (2011). GRADE guidelines: a new series of articles in the Journal of Clinical Epidemiology. J Clin Epidemiol.

[40] Patil AK, Keluskar K, Gaitonde S (2005). The clinical application of prostaglandin E1 on orthodontic tooth movement-A clinical trial. J Indian Orthod Soc.

[41] Ciur M-DI (2016). Evaluation of the influence of local administration of vitamin D on the rate of orthodontic tooth movement. Med-Surg J.

[42] Yussif NMA (2018). Efficacy and safety of locally injectable vitamin C on accelerating the orthodontic movement of maxillary canine impaction (oral mesotherapy technique): prospective study. Clin Cases Miner Metab.

[43] Varughese ST (2019). Effect of vitamin D on canine distalization and alveolar bone density using multi-slice spiral CT: a randomized controlled trial. Dent Pract.

[44] GRADEpro, G. GRADEpro guideline development tool [software]: McMaster University; 2015.

[45] Proffit WR, et al. Contemporary orthodontics-e-book: Elsevier Health Sciences; 2018.

[46] Krishnan V, Davidovitch Ze (2006). Cellular, molecular, and tissue-level reactions to orthodontic force. Am J Orthod Dentofacial Orthop.

[47] Henneman S, Von den Hoff J, Maltha J (2008). Mechanobiology of tooth movement. Eur J Orthod.

[48] Turner AG, Anderson PH, Morris HA (2012). Vitamin D and bone health. Scand J Clin Lab Invest.

[49] Boyle WJ, Simonet WS, Lacey DL (2003). Osteoclast differentiation and activation. Nature.

[50] Mori K (2006). Modulation of mouse RANKL gene expression by Runx2 and PKA pathway. J Cell Biochem.

[51] St. John HC (2014). The osteoblast to osteocyte transition: epigenetic changes and response to the vitamin D3 hormone. Mol Endocrinol.

[52] Triliana R (2016). Skeletal characterization of an osteoblast-specific vitamin D receptor transgenic (ObVDR-B6) mouse model. J Steroid Biochem Mol Biol.

[53] Han G (2004). Expression of cathepsin K and IL-6 mRNA in root-resorbing tissue during tooth movement in rats. Zhonghua Kou Qiang Yi Xue Za Zhi.

[54] Bumann A (1997). Collagen synthesis from human PDL cells following orthodontic tooth movement. Eur J Orthod.

[55] Rodriguez IA, et al. Platelet-rich plasma in bone regeneration: engineering the delivery for improved clinical efficacy. Biomed Res Int. 2014;2014. 10.1155/2014/392398 PMC4094865 25050347

[56] Anitua E (2015). Leukocyte inclusion within a platelet rich plasma-derived fibrin scaffold stimulates a more pro-inflammatory environment and alters fibrin properties. PLoS One.

[57] Kang Y-H (2011). Platelet-rich fibrin is a bioscaffold and reservoir of growth factors for tissue regeneration. Tissue Eng Part A.

[58] Otsuka E (2000). Role of ascorbic acid in the osteoclast formation: induction of osteoclast differentiation factor with formation of the extracellular collagen matrix. Endocrinology.

[59] Tsuneto M (2005). Ascorbic acid promotes osteoclastogenesis from embryonic stem cells. Biochem Biophys Res Commun.

[60] Litton SF (1974). Orthodontic tooth movement during an ascorbic acid deficiency. Am J Orthod.

[61] Dreizen S, Levy BM, Bernick S (1969). Studies on the biology of the periodontium of marmosets: VII. The effect of vitamin C deficiency on the marmoset periodontium. J Periodontal Res.

